# VIRTUAL IMPLEMENTATION OF MOBILITY PROGRAMS IN MEDICALLY COMPLEX POPULATIONS

**DOI:** 10.1093/geroni/igae098.0706

**Published:** 2024-12-31

**Authors:** Alexander Garbin, Jennifer Stevens-Lapsley

**Affiliations:** Chair; Co-Chair

## Abstract

Utilization of virtual platforms (e.g., Zoom, Teams, VA Video Connect) has rapidly expanded in both clinical and research settings since the onset of the COVID-19 pandemic. Within research, the use of virtual platforms affords numerous advantages including access to a broader geographical range of older adults, lack of dependency on transportation, and ability to monitor fidelity remotely. However, there also exist numerous considerations to ensure success when using virtual platforms as part of a research study. This symposium will detail four research projects utilizing virtual platforms. The research projects discussed share the overarching theme of improving mobility in older adults with medical complexity while varying in their stages of research, methodologies used, and how they use virtual platforms. The first speaker (Ritchey) will detail implementation of a virtual fall prevention clinical trial, MOVing FREEly. The second speaker (Garbin) will present on the use of a virtual platform to 1) determine the prevalence of fall risk factors in older Veterans using virtual fall risk factor assessments and 2) examine Veterans’ and providers’ perceptions of virtual fall prevention strategies. The third speaker (Rauzi) will discuss the development and refinement of a multicomponent telerehabilitation intervention delivered via virtual platform to older Veterans, using mixed-methods approaches. The fourth speaker (Stevens-Lapsley) will discuss challenges and successes of using virtual platforms and technologies to remotely monitor fidelity in a large-scale pragmatic clinical trial. These talks will provide a comprehensive understanding of barriers, solutions, and considerations for employing virtual platforms across a range of studies.

